# A randomised controlled trial of interventions for taxane-induced nail toxicity in women with early breast cancer

**DOI:** 10.1038/s41598-022-13327-6

**Published:** 2022-07-07

**Authors:** Audrey Morrison, Rebecca Marshall-McKenna, Angus K. McFadyen, Cathy Hutchison, Ann-Marie Rice, Lynne Stirling, Pauline McIlroy, Iain R. Macpherson

**Affiliations:** 1 The Beatson West of Scotland Cancer Centre, 1053 Great Western Road, Glasgow, G12 0YN Scotland; 2 School of Medicine, Dentistry and Nursing, University of Glasgow, Glasgow, G12 8QQ Scotland; 3 AKM-Stats, Glasgow, Scotland; 4 Institute of Cancer Sciences, University of Glasgow, Garscube Estate, Switchback Road, Glasgow, G61 1QH UK

**Keywords:** Cancer, Health care, Oncology

## Abstract

Onycholysis and paronychia has been associated with chemotherapy treatment for women with breast cancer. Our primary aim was to investigate the effectiveness of different topical interventions to ameliorate nail toxicity. Secondary aims were to explore the full range and severity of possible nail changes associated with taxane-based chemotherapy and the specific impact this had on quality of life, using two novel measures. This was an exploratory randomised controlled trial of three topical interventions (standard care, nail polish or specialist nail drops) for the prevention or reduction of nail changes induced by taxane-based chemotherapy. Outcomes included nail toxicity assessed at three time points (baseline, 3 weeks and 3 months post completion of chemotherapy) using two novel clinical tools (NToX-G12, NToX-QoL) and the Common Terminology Criteria for Adverse Events (CTCAE v3) and EQ-5D-5L. A total of 105 women were recruited (35 in each arm) and monitored up to three months post completion of chemotherapy. Almost 20% of patients were over the age of 60 years. There were 26 withdrawals, the majority from the nail polish arm. Residual Maximum Likelihood REML analysis indicated a significant arm, time and interaction effect for each intervention (p < 0.001). Less nail toxicity was observed in patients receiving specialist nail drops or standard care arms in comparison to those using nail polish. This study provides evidence to support clinicians’ suggestions on nail care recommendations based on the patients’ needs and preferences. Future investigations into comparing or combining cryotherapy and topical solutions that can support patient’s decisions are warranted.

## Introduction

Taxane-based chemotherapy regimens have improved disease-free and overall survival rates in early breast cancer (EBC)^1^, yet cause cutaneous toxicities including skin, hair and nail changes^2^. Chemotherapy-related nail changes occur in 34.9% of patients receiving docetaxel and 43.7% of patients receiving paclitaxel^3^. Many patients are less aware of nail toxicity as an adverse reaction of treatment^3^. However, those who do know are anxious about how changes will affect the appearance of their nails^4^. Adverse effects of taxane chemotherapy may include physical impact e.g., pain, functional impairment and/or a psychological impact on altered body image and well-being^5–9^.

Nail changes may occur on the fingernails or toenails. Nail changes are a result of direct cytotoxic effects on the matrix cells, affecting the nail plate, nail bed, blood vessels and periungual tissue^4^, thus may vary in appearance, severity and impact on quality of life (QoL)^4,8^. Fingernails have an average growth rate of 3 mm per month and changes tend to become apparent following numerous chemotherapy cycles, increasing with cumulative dose^3^ and with a possible contribution of the formulation vehicles cremophor EL and Tween 80^10^.

Previous studies have focused on onycholysis and paronychia^11–13^, but often lack comprehensive information about the full spectrum of nail changes that may be experienced^10,14,15^. The variation and lack of reporting of nail changes in large clinical trials has led to calls for standardised evaluation instruments^3^. Indeed, there is inconsistency in grading of dermatologic adverse events associated with cancer treatment between clinicians and patients when using the Common Terminology Criteria for Adverse Events (CTCAE), the most widely used instrument in clinical research settings^16,17^.

Various approaches have been investigated to ameliorate taxane-induced onycholysis and cutaneous toxicity including cryotherapy with the use of frozen gloves and socks^18,19^. Although usually well-tolerated, some patients are unable to cope with cryotherapy devices due to cold intolerance or problems with venous access^20,21^. A recent systematic review of preventative interventions for taxane-induced dermatologic adverse events reported that cryotherapy gloves and socks may be associated with reduced nail and skin changes but highlighted the need for larger and more standardised randomised clinical trials^22^. Related interventions such as cooling gel pads^23^ and cold water immersion^24^ may also be beneficial but lack robust evidence.

Topical solutions have also been investigated with one randomised controlled trial (RCT) reporting significant reductions in docetaxel -induced onycholysis using a hydrating nail solution (HNS)^11^. Polyphenolic-rich nail balm has also demonstrated reduced nail damage, albeit in a small, uncontrolled trial^12^. Improvements in paronychia with use of a 2% povidone-iodine solution were reported in a small retrospective case series^13^. Persisting anecdotal evidence from patients suggest the benefits of wearing dark or black coloured nail polish despite no clinical evidence to support this. Therefore, our primary aim was to evaluate the effectiveness of different topical interventions to reduce the incidence and minimise the severity of chemotherapy-induced nail problems in women receiving taxane-based chemotherapy for EBC. Secondary aims were to investigate the severity of nail problems, and any associated impact on QoL, using two novel nail toxicity outcome measures.

## Methodology

### Study design

This phase II, exploratory randomised controlled trial (RCT) evaluated interventions to prevent or minimise nail changes associated with taxane treatment, aiming to capture the extent and severity of all nail changes in female patients diagnosed with breast cancer (BC). The study is registered on www.clinicaltrials.gov (22/10/2015: NCT02583204).

### Recruitment

From January 2017 to July 2018, women aged 18 years or over, diagnosed with EBC, were recruited at the Beatson West of Scotland Cancer Centre (BWoSCC) and satellite clinics in NHS Greater Glasgow and Clyde. Inclusion criteria were patients due to commence standard neo-adjuvant or adjuvant taxane-based chemotherapy regimens including FEC-D (5 fluorouracil 500 mg/m^2^/epirubicin 100 mg/m^2^/cyclophosphamide 500 mg/m^2^ followed by docetaxel 100 mg/m^2^ q21 × 3 cycles), or TC (Taxotere 75 mg/m^2^/Cyclophosphamide 600 mg/m^2^ q21 × 4 cycles). Patients with HER2-positive EBC received trastuzumab concomitant with the taxane. Exclusion criteria included previous treatment (< 2yrs) with taxane-chemotherapy; known hypersensitivity to nail products; artificial nails or shellac (< 3 months); neurological deficit to upper limb. Patient preferences, usual nail care routine, expectations, and lifestyle factors that may interfere with protocol adherence were explored prior to recruitment. It was emphasised that no other product could be used on the nails other than the assigned intervention for the duration of trial participation. The study was conducted in compliance with Good Clinical Practice guidelines and was approved by the South East Scotland Research Ethics Committee 02 (15/SS/1072). Clinicaltrials.gov identifier (NCT02583204).

### Interventions

Standard care included lifestyle and hand hygiene advice to prevent infection and damage to the nails; e.g., wearing household gloves when using chemicals, washing up or gardening; to file nails rather than cutting, to moisturise hands and regularly apply simple moisturising cuticle oil around fingernails.

Two interventions were compared with standard care. Firstly, taxane-induced nail changes may be precipitated by sunlight or UV exposure^25^, thus the use of a nail covering may provide some protection. Despite the lack of evidence, painting nails with dark nail varnish has been a popular method of preventing cancer related nail problems on patient blogs and online sites^26,27^.

The second intervention OnicoLife^®^ is a nail-specific medical device to treat tender and fragile nails in patients undergoing chemotherapy treatment. This intervention contains a specific Fatty Acids Group and the amide PEA, which help control mast cells and macrophage re-activity and has anti-inflammatory, antibacterial and antifungal properties (https://www.againlifeitalia.com/en/f-a-g-and-research).

### Procedure

All participants were given the same routine lifestyle, hand hygiene and nail care advice. Randomisation was performed using random permuted sequenced blocks to incorporate both intervention and site. Envelopes containing the next single sequence permutation were selected by the participant to reveal allocation to one of three arms comprising of *Standard Care* (SC), *Nail Polish* (NP) or *OnicoLife Drops*® (OD).

In the SC arm, 1–2 drops of Skintruth cuticle oil were applied twice daily to each nail, massaged in with small circular motions. In the OD arm, 1–2 drops were similarly applied and massaged. In the NP arm, participants were asked to apply and maintain polished nails using a nail polish containing no toluene, dibutyl phthalate or formaldehyde (China Glaze®). To prevent nail staining and hypersensitivity reactions, the gold standard method of application was used (one base coat, two coats of colour, and one top coat). For removal/reapplication of polish, participants were provided non-toxic, non-acetone nail polish remover with baby wipes, rather than cotton wool, to prevent snagging damage to the nails by cotton fibres. Top / base coat, and remover were also from the Skintruth product range. Each participant received an explanation about the assigned intervention. Participants in the NP arm were additionally shown a brief video clip to ensure consistency of application.

### Outcome measures

There are no existing tools to capture nail toxicities and the full range of possible nail changes associated with taxane-based treatment. The CTCAE version 3 was routinely used in breast clinics. Thus, we designed two novel instruments to support the study endpoints. These instruments were developed in the oncology setting with a multi-disciplinary team and collaborative NHS/Academic Breast Cancer Research Group, and externally reviewed by expert clinicians/academics (e.g., oncology/ podiatry/psychology). The instruments were piloted with staff and patients in daily practice before commencing the RCT. The instruments/assessments were completed in a real time clinical environment with a team of five chemotherapy nurses and three breast clinical nurse specialists (CNS).

The NToX-G12 (clinical assessment), incorporated an assessment sheet and master visual guide, matching each item on the nail assessment form to a photograph. The presence and severity of each of the 12 items were assessed and weighted according to extent of nail damage and number of fingernails involved. The maximum score was 524, with higher scores indicating increased severity.

The accompanying NToX-QoL (quality of life measure) was developed to specifically measure changes in quality of life associated with nail changes for this RCT. This 18-item self-report questionnaire was compared with the EQ-5D-5L used in clinical practice. Both incorporate physical, functional, and emotional domains and a visual analogue rating scale.

The details of the acceptable psychometric properties of both the NTox-G12, and NToX-QoL (not reported here) have been presented^30,31^. Full details of these tools are the subject of a separate manuscript and are available from the authors on request pending publication.

### Study assessments

The study duration was approximately 30 weeks from commencement at baseline assessment (dependant on treatment delays during chemotherapy cycles). Assessments were conducted at three time points; (T1) baseline (prior to second cycle of chemotherapy); (T2) and (T3) were three weeks and three months post-administration of the final cycle of chemotherapy.

At each assessment all fingernails were examined and the severity of nail changes recorded using the NToX-G12 and CTCAE (v3). Relevant clinical information such as changes to treatment regimen, dose reduction, medication changes, or any relevant blood test results outside normal parameters were also documented. Medical photography provided a visual record of nail changes over all three study time points.

### Statistical analysis

Based on estimates in the variability of the main outcome measure (NToX-G12), obtained during previous validation of the tool, we estimated that a power of 80% could be achieved at the 5% level of significance if 25 participants were recruited in each study arm. To allow for dropouts and other extraneous factors, the aim was to recruit 35 women in each arm.

Analysis was performed using IBM SPSS v 22.0. Descriptive statistics and Spearman’s correlation were used to summarise each variable and relationships between variables. Given the higher than expected dropout levels, Linear Mixed Models with Restricted Maximum Likelihood (REML) was used to determine the effects of each factor within each model. Post hoc analysis with correction factors was used, where appropriate to assess the Time effects between T1 and T2, T1 and T3 to seek out when nail changes occurred in nail condition(s) and QoL. Main effects and interaction effects were inspected for significance and where no statistical significance was identified, plots of estimated means over time were constructed in order to seek out any clinical relevance.

### Informed consent

Informed consent for all participants was obtained for medical photography at each study visit, and separate consent obtained for use of anonymised personal quotes and nail images for publication purposes. Images contained in this publication are only from those who provided this level of consent.

### Conference presentation

This study and the outcome measures developed were presented as posters at the 1st UK Interdisciplinary Breast Cancer Symposium (January 2018, Manchester) https//doi.org/ 10. 1007/s10549-017–4585-x and the UK Oncology Nursing Society annual conference (November 2015, Birmingham; November 2018, Glasgow) and as an oral presentation at the Scottish Cancer Trials Breast Cancer Group Meeting (November 2018, Perth).

## Results

Of 180 potentially eligible patients approached, 105 were recruited to the trial (35 in each arm). Participant characteristics are displayed in Table 1. The most commonly received systemic anti-cancer treatment was FEC-D (FEC-D, 71.4%: TC, 28.6%). Approximately one-third of participants (n = 37; 35.2%) also received Trastuzumab treatment in addition to their prescribed taxane regime. The majority had no pre-existing health issues with only 25% reporting co-morbidity. In keeping with the known toxicity profile of docetaxel, almost 40% (n = 38) required a treatment dose reduction (Table 2).

**Table 1:** Summary of participant characteristics.

Demographics									
Characteristic	Category	Results							
Age	Under 60 yrs	84 *(80%)*							
	Over 60 yrs	21 *(20%)*							
	Range (Median)	29 – 77 years (52yrs)							

Characteristic	Category	Standard Care SC (n = 35)		Nail Polish NP (n = 35)		OnicoLife® OD (n = 35)		Total ** (n = 105)	
		n	%	n	%	n	%	n	%
Age range 29–77 yrs median 52 yrs	< 60 years	27	32.1	31	36.9	26	31.0	*84*	80.0
	> 60 years	8	38.1	4	19.0	9	42.9	*21*	20.0
Ethnicity	White British	15	42.9	15	42.9	19	54.3	49	46.7
	White Scottish	17	48.6	18	51.4	16	45.7	51	48.6
	White-Other	1	2.9	1	2.9	0	0	2	1.9
	Asian/Asian British	0	0	1	2.9	0	0	1	1.0
	Black/Black British-African	2	5.7	0	0	0	0	2	1.9
Regime	TC	8	22.9	8	22.9	14	40.0	30	28.6
	FEC-D	27	77.1	27	77.1	21	60.0	75	71.4
Treatment	Adjuvant	22	62.9	21	40.0	29	82.9	72	68.6
	Neo-adjuvant	13	37.1	14	40.0	6	17.1	33	31.4
Trastuzumab	Yes	13	37.1	8	22.9	15	42.9	36	35.2
	No	21	60.0	27	77.1	20	57.1	68	64.8
Smoker status	Smoker	4	11.4	6	17.1	2	5.7	12	11.4
	Ex-smoker	3	8.6	4	11.4	6	17.1	13	12.4
	Non-smoker	26	74.3	21	60.0	23	65.7	71	67.6
Comorbidity	Present	5	14.3	9	25.7	12	34.3	26	24.8
	Not present	29	82.9	26	74.3	21	60.0	76	72.4
Job status	Employed	21	60	24	68.6	23	65.7	68	64.8
	Unemployed/retired	14	40	11	31.4	12	34.3	37	35.2
Occupation If employed	Administration	5	23.8	10	41.6	5	21.6	20	19.0
	Professional/ academic	6	28.5	4	16.6	2	8.6	12	11.4
	Support work e.g., carer/housewife	3	14.3	3	12.5	4	17.3	10	9.6
	Management	2	9.5	2	8.3	4	17.3	8	7.7
	Sales/retail	3	14.3	2	8.3	2	8.6	7	6.7
	Hospitality	0	0	1	4.2	4	17.3	5	4.8
	Other	2	9.5	3	8.3	3	13.0	6	5.7

Significant values are in italics.

**Table 2:** Summary of alterations to treatment due to toxicity.

Characteristic	All		Standard Care		Nail Polish		OnicoLife ®	
	*n*	%	*n*	%	*n*	%	*n*	%
**Changes to original regime?**								
Yes	26	24.8	6	17.1	10	28.6	10	28.6
No	77	73.3	28	80.0	25	71.4	24	70.6
**Any dose reductions?**								
Yes	37	35.2	9	25.7	12	34.3	16	45.7
No	60	57.2	22	62.9	21	60.0	17	48.6
Missing	8	7.6	4	11.4	2	5.7	2	5.7
Dose reduction (Range) +			10–25%		10–30%		10–40%	

### Nail changes

**Figure 1:**
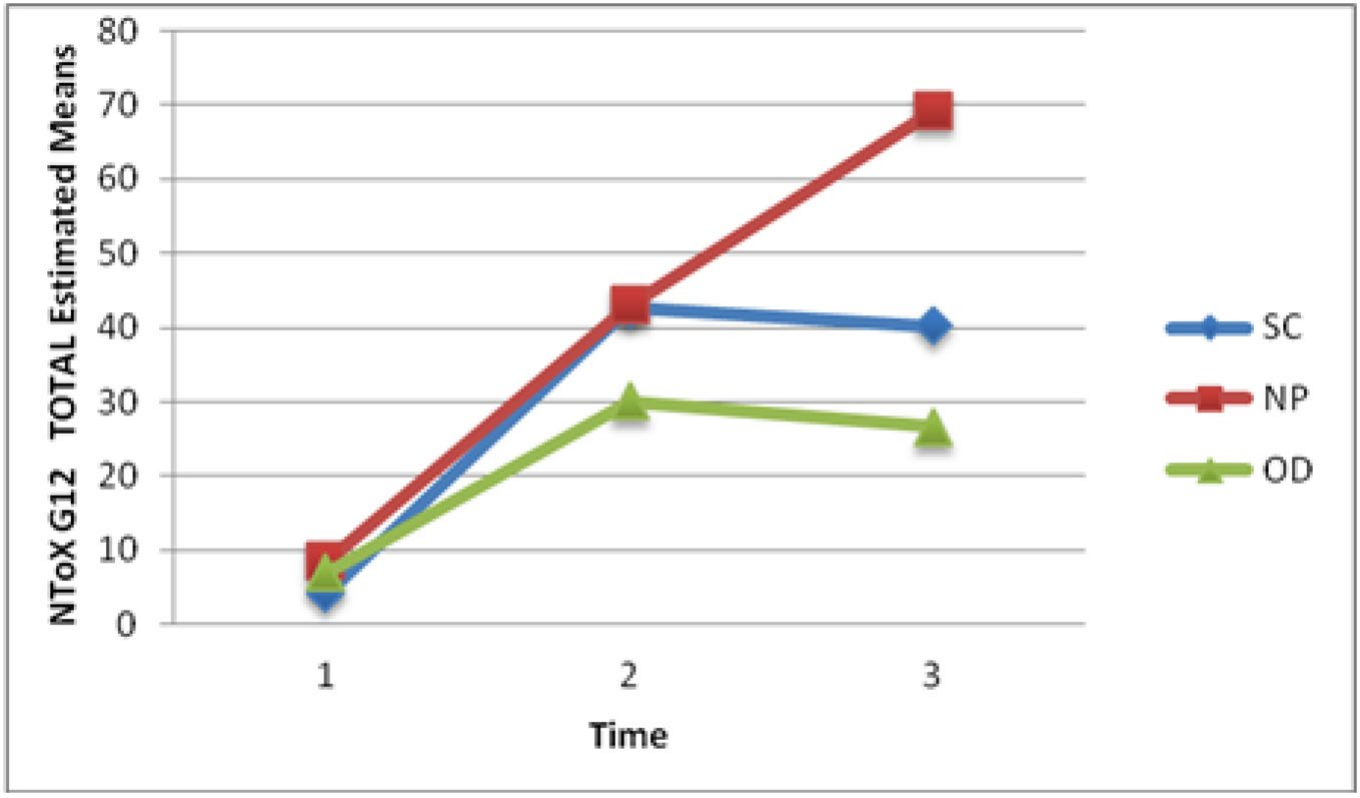
Pattern of change in NToX-G12 mean scores over time by intervention.

The REML analysis of NTox-G12 scores (Fig. 1) indicated a significant arm effect, time effect and interaction effect (p < 0.001 for each). All estimated mean scores increased [indicating deterioration in terms of nail damage] between baseline and post chemotherapy visits. However, the SC and OD Arms tended to level off immediately after chemotherapy treatment ceased. In contrast, the NP Arm illustrated a further increase in mean up to the final follow up visit; hence there was a very significant interaction effect. Post hoc analyses clearly showed the significant Time effect between T1 and T2 [mean increase = 32.01, SE. = 3.02, 95% CI (24.63, 39.40), p < 0.001] and between T1 and T3 [mean increase = 38.77, SE. 3.31, 95% CI (30.66, 46.88), p < 0.001] but not between T2 and T3 [mean increase = 6.76, SE. 4.31, 95% CI (-3.78, 17.30), p = 0.362]. Similarly, significant Arm effects were found between NP and SC [mean difference = 11.22 SE. = 4.25, 95% CI (0.86, 21.60), p = 0.029] and between NP and OD [mean difference = 19.00, SE. = 4.09, 95% CI (9.00, 28.99), p < 0.001]. All post hoc p-values were adjusted for multiple comparisons. Given this clear difference in mean score at final study visit (T3) it should however be stated that these means of approximately, 27, 40 and 69 are all in the lower range of the NToX-G12 scale which has a maximum score of 524.

**Figure 2:**
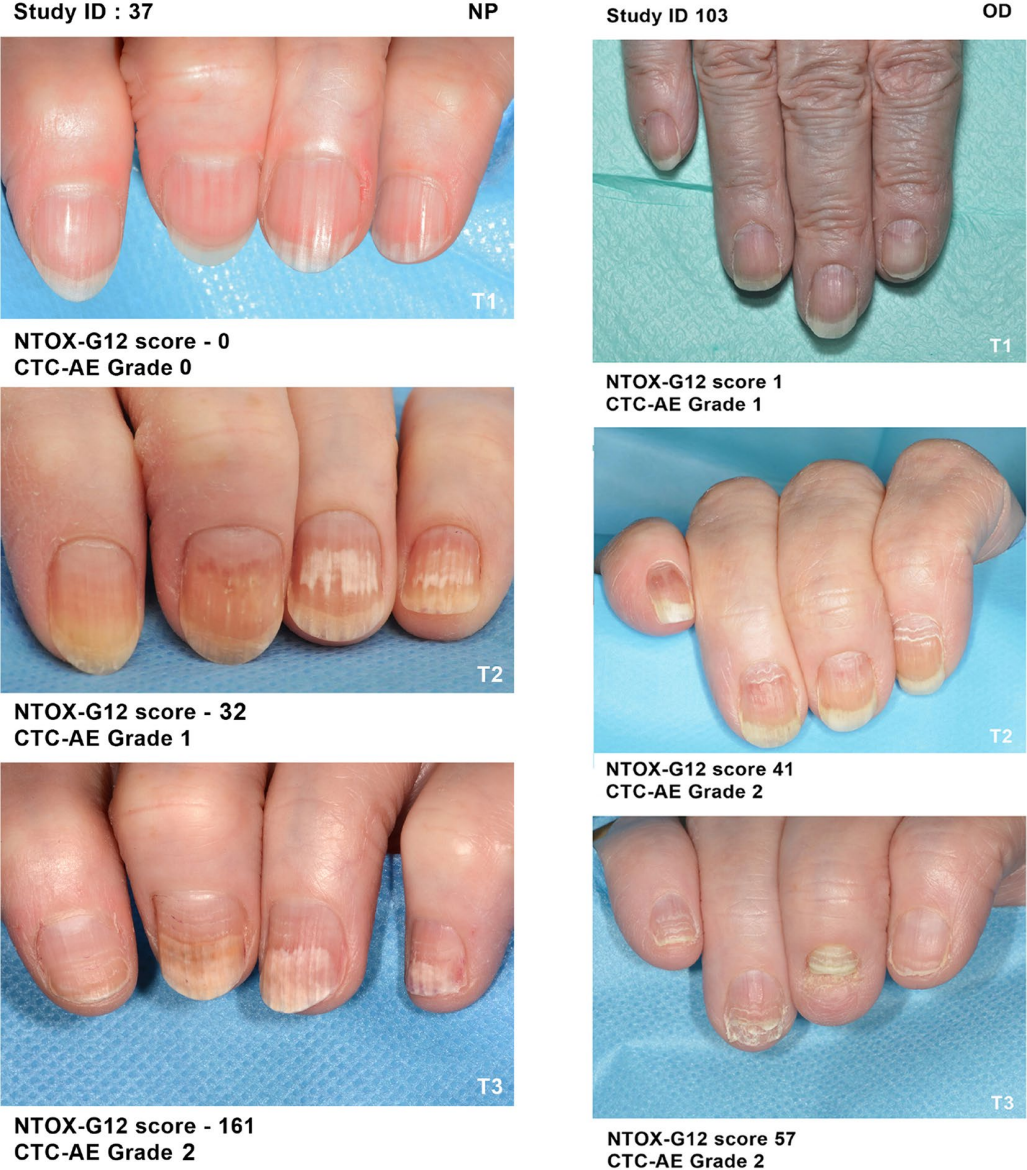
Examples of progression of nail changes over time (arranged in order top to bottom: baseline, 3 weeks pre-, and 3 months post-chemotherapy).

**Figure 3:**
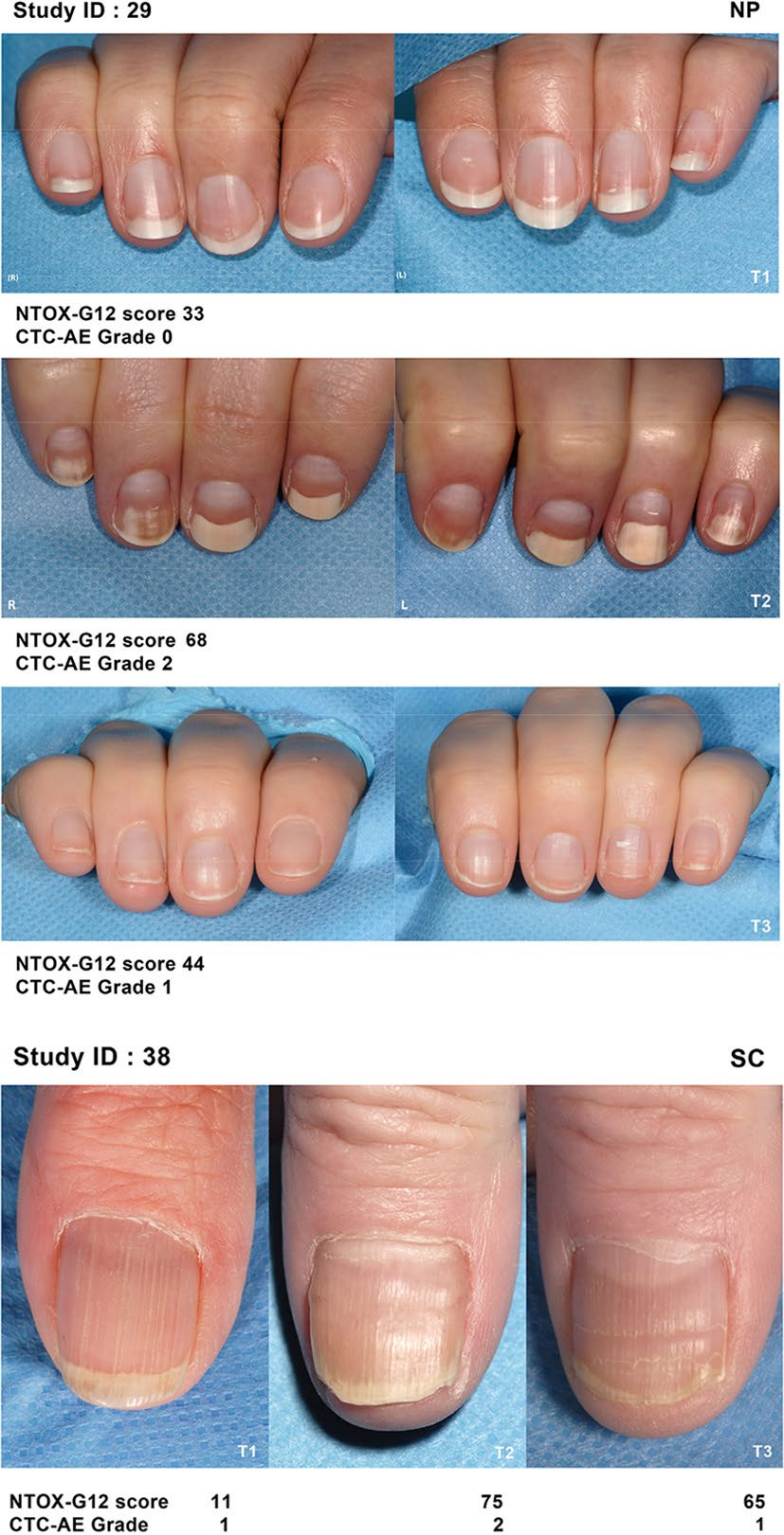
Examples of resolution of nail changes over time (T1-T3).

**Figure 4:**
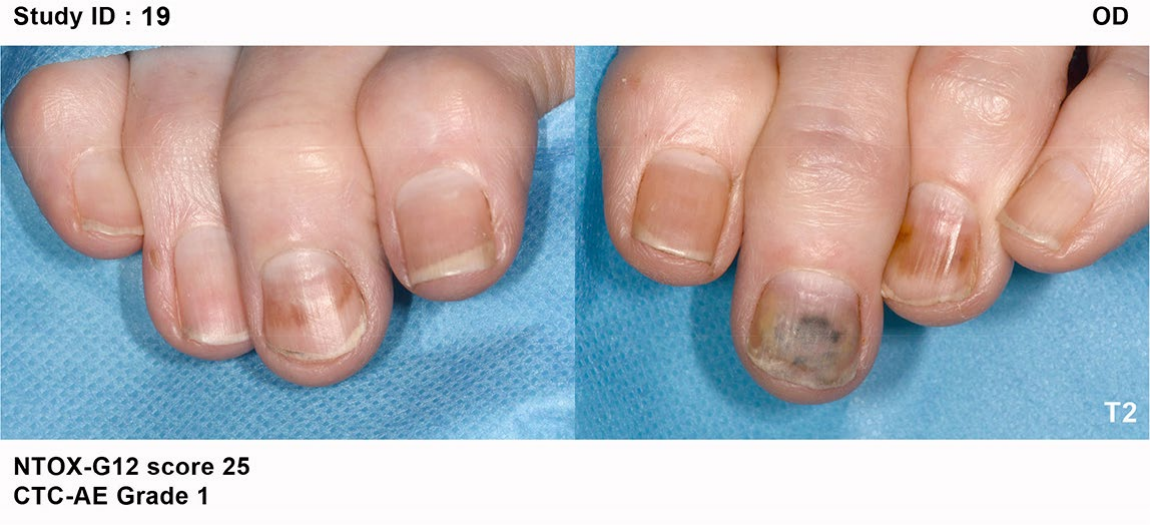
Variation/ pattern of changes in affected fingernails of right and left hand.

At baseline (T1) the majority of patients had no evidence of pre-existing nail problems, motor or sensory neuropathy. All arms, reported an increase in severity with the most severe, on average, being the NP group and least severe the OD group. In terms of actual numbers, all patients in the NP group increased in severity. Whilst 7.7% of patients in the SC group exhibited no change, 3.8% of patients showed an improvement. In the OD group10% of patients exhibited no change yet 6.6% of patients had improvement of their nail condition. Medical photography captured the severity, progress (Fig. 2) and resolution (Fig. 3) of any nail changes at each study visit. Variation was noted in the appearance of nail changes (Fig. 4) and the number of fingernails affected (Table 3).

**Table 3:** Mean number of nails affected per nail problem.

Mean number of fingernails affected by category			
Nail Issue	Study Time Points		
	T1	T2	T3
Pitting	4.6	5.2	5.2
Smooth ridging	2.0	5.5	5.5
Rough ridging	2.5	5.3	5.5
Redness/ inflammation	3.8	3.0	4.0
Hard skin build-up	4.8	4.1	4.2
Skin hacks/ breaks	2.5	2.6	3.2
Discolouration- white	4.0	5.5	5.2
Discolouration-yellow	1.5	5.1	5.2
Discolouration- brown/black	5.5	5.6	4.4
Brittleness	4.0	4.0	5.1
Splinter haemorrhage	2.0	2.6	2.5
Infection	0.0	0.0	0.0
Partial loss	1.0	5.0	4.7
Complete loss	0.0	3.0	0.0

**Figure 5:**
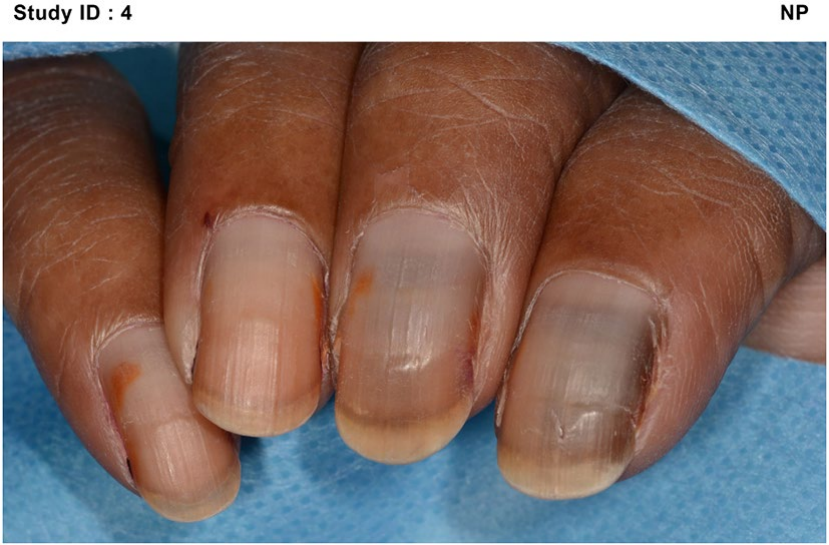
Nail discolouration (orange): ethnic factors.

In addition to the NTox-G12’s measure of the number of nails affected by each separate item, photography provided supporting visual evidence and a permanent record of nail toxicity. The second visit, when most nail changes first occurred, the most common changes were pitting, ridging, brittleness and white discolouration of the nail. Most tended to resolve except for those using nail polish, which had more persistent yellow discolouration than any of the other arms. In the three participants representing Asian/ Black ethnicity, orange discolouration was more common. Clinicians reported difficulty grading the nails of these three women, especially in distinguishing discolouration (Fig. 5).

The NToX-G12 scores were compared with grades on the CTCAE. At baseline, 23 out of 105 participants showed Grade 1 changes. In comparison, the NT0X-G12 identified nail changes in 72 out of 105 participants with total scores ranging from 1–38. No significant difference was found at either baseline or three weeks post-chemotherapy (T2) timepoints. However, at the final visit (T3) there was a significant difference in mean scores for those with Grade 1 on the CTCAE (Table 4). This showed nail polish being significantly different from both Standard Care (p = 0.011) and OnicoLife Drops® (p = 0.001). However, no such difference was noted for those with Grade 2 scores, although it should be noted that samples with a grade 2 score were very small.

**Table 4:** Comparison of mean scores for NTOX-G12 and CTCAE.

Intervention arm	CTC grade time 3		
	Absent	Grade 1	Grade 2
**NToX-G12 score**			
**Standard care [SC]**			
Mean	2.3	34.4	82.6
SD	4.0	9.0	38.1
Median	0	31.5	84.0
**Nail polish [NP]**			
Mean	–	51.5	99.9
SD		15.3	42.0
Median		50.0	87.0
**OnicoLife® drops [OD]**			
Mean	6.7	31.1	70.0
SD	9.5	17.6	18.4
Median	4.0	30.0	70.0

Overall, 26 participants dropped out of the trial. Nine dropped out from the SC arm (25.7%), 13 from the NP arm (37.1%), but only four subjects (11.4%) dropped out of the OD Arm. The main reason for dropout was chemotherapy toxicity with three participants requiring acute hospitalisation as a result of treatment toxicities. Other reasons related to issues experienced with the assigned intervention e.g., aversion to the smell or colour of the intervention.

The NToX-QoL showed no statistical difference over the three time points. However at time 3 (end of study) the NToX-QoL physical subscale and the EQ-5D-5L subscale for pain and discomfort showed significant Spearman’s Rho correlation (− 0.438. p = 0.025) at the 0.05 (two-tailed) level.

### End of study evaluation

A patient reported evaluation questionnaire on the experience of using the assigned product revealed a very small number, in each intervention arm, reported brief periods of non-compliance with the protocol, while some missed applications, typically for a few days post-infusion or due to surgery and general lethargy. Based on participants completing the study (62/79, 78.5%), the majority reported their assigned intervention helped to mitigate nail changes (Table 5).

**Table 5:** End of trial evaluation of assigned intervention.

Intervention	End of Study Feedback
Standard Care (SC)	I did not like the oil. It did moisturise my nails but did not help with the soreness I had
	I bite all my nails but now have lovely, long nails
	Made my nails a lot stronger than normal and I found they grew quicker
	It has made me more aware of my nails and how to look after them
	The oil helped prevent nail loss- one less worry during treatment
Nail Polish (NP)	My finger nails were at their worst about six weeks after end of treatment
	I didn’t have too many effects from chemo but my nails are in a bad way- Nail polish is still useful in hiding my (nail) condition
	Nails became very discoloured and weak; a number of them lifted and three snapped off
	Made me more aware of taking care of my nails during chemotherapy
	Felt the nail polish helped to strengthen my nails
	I find it difficult to open a can with the ring pull and to open packages
OnicoLife ®Drops (OD)	Although my nails were damaged somewhat throughout my treatment they have recovered quickly but I have also noticed my toenails which I kept painted with a dark nail polish throughout are in a really bad condition in comparison
	I noticed no difference in my fingernail condition during treatment compared to my toenails which *are* affected
	My toenails fell off whereas my fingernails did not
	I was delighted with the results of my nails, I used the nail drops religiously every day and it helped me feel I was in control My nails were stronger than usual and I intend to carry on with it
	The OnicoLife drops have really helped
	It is hard to know exactly how my nails would have been if not involved in this study, but I am very pleased how my nails have been in comparison to other people receiving the same treatment

## Discussion

To our knowledge, this was the first RCT that investigated a range of topical interventions for taxane-induced nail changes in women with EBC whilst providing standard care advice on wearing gloves, maintaining hand hygiene and nail care, avoidance of toxic ingredients and artificial nail use. The results suggest that combined with guidance on hand hygiene and nail care, both the specialised OnicoLife ® nail drops and nail oil were associated with less nail toxicity than use of dark nail varnish. Despite asking patients to comply with the protocol before making their decision on participation, a slightly higher dropout than anticipated occurred in the NP intervention arm.

Additionally, the effectiveness of the interventions were monitored three months from the date of the participant’s final infusion, thus providing time to appraise nail growth and condition over the longer term. Therefore, longer than the commonly reported follow up period in other studies^11–13^.

Owing to the greater number of categories incorporated in the NToX-G12 assessment of nails, our study also identified pre-existing nail issues at T1 (baseline). This is in keeping with other studies^11^. However, most of these were minor issues common to healthy individuals such as hard skin and skin hacks. Similarly, at T2, the most common and persistent changes were ridging (Beau’s lines) pitting, brittleness and white nail discolouration. Orange nail discolouration has previously been linked with haemorrhagic suffusion of the nail bed^4^. In our study, it was not possible to ascertain why orange discolouration was only seen in fingernails of women from certain ethnic origins, since all participants recruited to the nail polish arm applied a base coat to prevent staining from nail polish. This may be worthy of exploration in future studies. No other dermatological concerns associated with nail products were reported.

The documentation of nail changes continues to be limited by the lack of a standardised instrument, thus leading to difficultly in comparing outcomes of previous nail interventions. The CTCAE has been previously criticised and grading of nail changes can only ever depend on the interpretation of the investigator. For example, only one oncology nurse specialist assessed all participants for nail toxicities using the CTCAE v4 in the RCT using HNS^11^, and in the polybalm study^12^ it was unclear how many physicians were involved in nail assessments or their area of expertise. In a real world oncology setting, consistent assessment by the same health care professional is often unrealistic; hence in our study we used a team of experienced chemotherapy and CNS nurses.

Although we addressed the development of two nail instruments for use in a clinical setting, the resulting NToX-G12 scores were in the lower range of the scale. As there were limited reports of more severe, nail changes with higher weighting such as moderate pain, infection, paronychia, or onycholysis, the relative homogeneity of the patients included in this trial may have affected the results.

To date, there have been various topical interventions investigated for the prevention of taxane associated nail changes^11–13^, which have varied in methods of application for example, brushing or painting the nail. In our study, participants using both the nail oil and specialised drops experienced less nail problems than those using nail polish. Since both interventions were applied by manually massaging the product onto the nail area^28^, we postulate it may be the massage action stimulating the vascular elements of the nail and nail matrix, thus helping ameliorate nail changes. This is in contrast with previous studies using cryotherapy, which suggest a reduction in vascular flow reduces drug exposure thus resulting in less toxicities^29^. If this is the case, it should be considered whether it is not only the topical agent, but also the method of massaging the agent into the nail that brings about effective change, which may be assisting in lesser occurrence/severity and overall better nail health. Thus, this is a suggested area for future investigation.

The cost of any intervention needs consideration when deciding to adopt into clinical practice, especially when resource and financial savings predominate. The purchase of multiple medical devices such as frozen gloves involves a substantial initial purchase, maintenance costs plus replacement of disposable single-use components. In addition, cryotherapy involves an additional burden on healthcare resource, whereas this is the opposite for self-managed interventions.

While no statistical difference was demonstrated for quality of life over the three time points, it is suggested to have clinical significance in that none of the study interventions demonstrated superiority in affecting quality of life. This is to be the subject of a separate paper for publication.

In conclusion, the results of this trial may support clinicians to suggest alternatives for patients based on their needs and preferences and advise nail oil or specialised drops may be better than the use of dark nail polish during and several months after taxane treatment for breast cancer.

While other studies involve only ‘cosmetic’ interventions, OnicoLife ® drops, as a nail- specific medical device has shown potential to reduce nail changes and thus, may be recommended to patients. Future investigation involving larger powered, RCTs into the comparison / combination of cryotherapy and topical solutions using suitably sensitive and specific outcome measures are warranted.

## Supplementary Information

Supplementary Information 1.

Supplementary Information 2.

Supplementary Information 3.
