# Supplementary material for: A randomised controlled trial of interventions for taxane-induced nail toxicity in women with early breast cancer
Source: Sci Rep. 2022 Jul 7;12:11575. doi: 10.1038/s41598-022-13327-6 (PMC9262963; doi:10.1038/s41598-022-13327-6)
Supplement: Supplementary file 1 — Supplementary Information 1. [file 41598_2022_13327_MOESM1_ESM.doc]

**Title: A randomised controlled trial of interventions for taxane-induced nail toxicity in women with early breast cancer**

Authors: Audrey Morrison^1^, [Audrey.Morrison@ggc.scot.nhs.uk](mailto:Audrey.Morrison@ggc.scot.nhs.uk); Rebecca Marshall-McKenna^2^, [Rebecca.Marshall @glasgow.ac.uk](mailto:Rebecca.Marshall @glasgow.ac.uk); Angus K McFadyen^3^, Akm-stats; [Akm@akm-stats.com](mailto:Akm@akm-stats.com); Cathy Hutchison^1^, [Cathy.Hutchison@ggc.scot.nhs.uk](mailto:Cathy.Hutchison@ggc.scot.nhs.uk); Ann-Marie Rice^2^, [Annmarie.rice@glasgow.ac.uk](mailto:Annmarie.rice@glasgow.ac.uk)

Lynne Stirling^1^, [Lynne.Stirling@ggc.scot.nhs.uk](mailto:Lynne.Stirling@ggc.scot.nhs.uk); Pauline McIlroy^1^, [Pauline.McIlroy@ggc.scot.nhs.uk](mailto:Pauline.McIlroy@ggc.scot.nhs.uk)

Iain R Macpherson^1, 4^, [Iain.Macpherson@glasgow.ac.uk](mailto:Iain.Macpherson@glasgow.ac.uk)

^1^The Beatson West of Scotland Cancer Centre, 1053 Great Western Road, Glasgow, G12 0YN.

^2^School of Medicine, Dentistry and Nursing, University of Glasgow, Scotland, G12 8QQ.

^3^AKM-Stats, Glasgow, Scotland.

^4^Institute of Cancer Sciences, University of Glasgow, Garscube Estate, Switchback Road, Glasgow, G61 1QH.

**Figure 1**

**Enrollment**

**Follow-Up /Withdrawal**

**(n=4)**

**Allocation**

**Follow-Up /Withdrawal**

**(n=9)**

**Follow-Up/Withdrawal**

**(n=13)**

Allocated to Standard Care

(n= 35)

Allocated to Nail Polish

(n=35)

health issues (n=4)

Assessed for eligibility (n=180)

Excluded (n= 75)

- declined to participate (n=27)
- lack of interest (n=21)
- other competing commitments (n=11)
- too anxious (n=16)

protocol non-compliance (n=9)

health issues (n=2)

excessive fatigue (n=1)

lethargy (n=1)

protocol non-compliance (n=6)

health issues (n=3)

lethargy (n=2)

lost to follow up (n=1)

change of treatment regime (n=1)

Randomized (n=105)

Allocated to OnicoLife® Drops

(n=35)
