# Supplementary material for: A randomised controlled trial of interventions for taxane-induced nail toxicity in women with early breast cancer
Source: Sci Rep. 2022 Jul 7;12:11575. doi: 10.1038/s41598-022-13327-6 (PMC9262963; doi:10.1038/s41598-022-13327-6)
Supplement: Supplementary file 2 — Supplementary Information 2. [file 41598_2022_13327_MOESM2_ESM.doc]

**Title: A randomised controlled trial of interventions for taxane-induced nail toxicity in women with early breast cancer**

Authors: Audrey Morrison^1^, [Audrey.Morrison@ggc.scot.nhs.uk](mailto:Audrey.Morrison@ggc.scot.nhs.uk); Rebecca Marshall-McKenna^2^, [Rebecca.Marshall @glasgow.ac.uk](mailto:Rebecca.Marshall @glasgow.ac.uk); Angus K McFadyen^3^, Akm-stats; [Akm@akm-stats.com](mailto:Akm@akm-stats.com); Cathy Hutchison^1^, [Cathy.Hutchison@ggc.scot.nhs.uk](mailto:Cathy.Hutchison@ggc.scot.nhs.uk); Ann-Marie Rice^2^, [Annmarie.rice@glasgow.ac.uk](mailto:Annmarie.rice@glasgow.ac.uk)

Lynne Stirling^1^, [Lynne.Stirling@ggc.scot.nhs.uk](mailto:Lynne.Stirling@ggc.scot.nhs.uk); Pauline McIlroy^1^, [Pauline.McIlroy@ggc.scot.nhs.uk](mailto:Pauline.McIlroy@ggc.scot.nhs.uk)

Iain R Macpherson^1, 4^, [Iain.Macpherson@glasgow.ac.uk](mailto:Iain.Macpherson@glasgow.ac.uk)

^1^The Beatson West of Scotland Cancer Centre, 1053 Great Western Road, Glasgow, G12 0YN.

^2^School of Medicine, Dentistry and Nursing, University of Glasgow, Scotland, G12 8QQ.

^3^AKM-Stats, Glasgow, Scotland.

^4^Institute of Cancer Sciences, University of Glasgow, Garscube Estate, Switchback Road, Glasgow, G61 1QH.

**NToX-G12 Nail Assessment Scale**

| **Study Participant ID:** | |  | | | | | | | | | | | |
| --- | --- | --- | --- | --- | --- | --- | --- | --- | --- | --- | --- | --- | --- |
|  | |  | | | | | | | | | | | |
| **Key:**  **A:** To indicate presence √, or absence 0  **B:** Enter number of nails affected.  **C:** Enter Total (**Score** x **B**) | | **Pre-assessment**  **Cycle 2** | | | | | | **Post-treatment 3 weeks** | | | **Post-treatment 3 months** | | |
|  |  | **Date:** | | | | | | **Date:** | | | **Date:** | | |
| **Description** | **Score** | **A** | B | | | **C** | | **A** | **B** | **C** | **A** | **B** | **C** |
| **1. Pitting** | 1 |  |  | | |  | |  |  |  |  |  |  |
| **2. Horizontal Ridging (Smooth)** | 1 |  |  | | |  | |  |  |  |  |  |  |
| **3. Horizontal Ridging (Rough)** | 1 |  |  | | |  | |  |  |  |  |  |  |
| **4. Skin around nails** |  | | | | | | | | | | | | |
| Red/ inflamed | 2 |  | |  | | |  |  |  |  |  |  |  |
| Hard skin build up | 2 |  | |  | | |  |  |  |  |  |  |  |
| **5.** **Skin breaks / Hacks** | 3 |  | |  | | |  |  |  |  |  |  |  |
| **6. Discolouration** |  | | | | | | | | | | | | |
| White | 3 |  | | |  | |  |  |  |  |  |  |  |
| Yellow | 3 |  | | |  | |  |  |  |  |  |  |  |
| Brown /black | 3 |  | | |  | |  |  |  |  |  |  |  |
| **7. Brittle nails** | 3 |  | | |  | |  |  |  |  |  |  |  |
| **8. Splinter Haemorrhage** | 4 |  | | |  | |  |  |  |  |  |  |  |
| **9. Infection** | 6 |  | | |  | |  |  |  |  |  |  |  |
| **10. Nail bed separation**   - Partial Loss | 8 |  | | |  | |  |  |  |  |  |  |  |
| **11. Nail bed separation**   - Total Loss | 10 |  | | |  | |  |  |  |  |  |  |  |
| **12. Pain in nail bed**  (Enter pain rating in column C) | None 0  Mild 2  Mod 4  Severe 6 |  | | |  | |  |  |  |  |  |  |  |
| **Total Score =**  (Add up rows 1-12 in Column C)  **Maximum Score = 524** |  |  | | | | |  |  | |  |  | |  |
| **Full name of Clinician:(Print)**  **Job Title:**  **Time taken to complete scale:** |  | _____ mins | | | | | | ____ mins | | | _____ mins | | |
