# Supplementary material for: A randomised controlled trial of interventions for taxane-induced nail toxicity in women with early breast cancer
Source: Sci Rep. 2022 Jul 7;12:11575. doi: 10.1038/s41598-022-13327-6 (PMC9262963; doi:10.1038/s41598-022-13327-6)
Supplement: Supplementary file 3 — Supplementary Information 3. [file 41598_2022_13327_MOESM3_ESM.pdf]

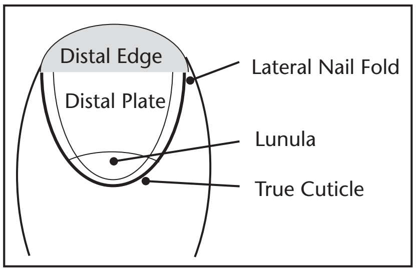

NHS
Greater Glasgow and Clyde

NToX-G12 - Nail Assessment Scale

Master Scoring Guide

Type of Changes	Example	Description	Column A<br>Weighted Score<br>(place ✔ in column if present)	Column B<br>Number of nails affected per relevant condition<br>Enter actual number of affected nails	Column C<br>Total Score =<br>Multiply score obtained in column A by score in column B and enter here
1. Pitting	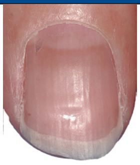	Visually apparent<br>Indent(s) felt on the nail plate surface	1 =YES		
2. Ridging (smooth)<br>Horizontal	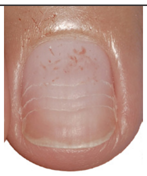	Visually apparent<br>Feels smooth to touch	1 =YES		
3. Ridging (rough)<br>Horizontal	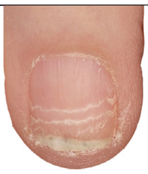	Visually apparent<br>Feels rough to touch	1 =YES		
4A. Skin around nails	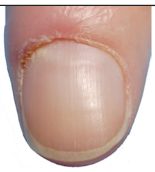	Red/ inflamed	2 = YES		
4B. Skin around nails	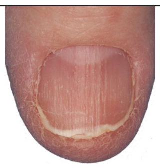	Hard skin build up	2 = YES		
5. Skin breaks /hacks	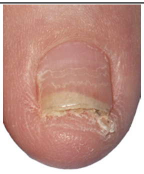	Open cut or crack in hardened skin around nail edge and / or nail fold	3 = YES		
6A. Discolouration	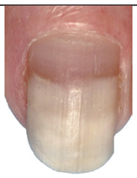	White	3 = YES		
6B. Discolouration	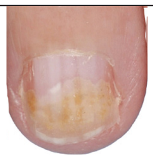	Yellow	3 =YES		

Ntox-G12 - Nail Assessment Scale

Master Scoring Guide

Type of Changes	Example	Description	Column A<br>Weighted Score<br>(place ✔ in column if present)	Column B<br>Number of nails affected per relevant condition<br>Enter actual number of affected nails	Column C<br>Total Score =<br>Multiply score obtained in column A by score in column B and enter here
6C. Discolouration	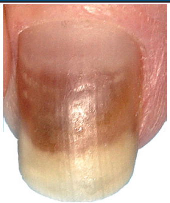	Brown/black	3 =YES		
7. Brittle nails	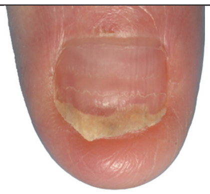	Nail plate breakage/ splitting	3 =YES		
8. Splinter haemorrhage	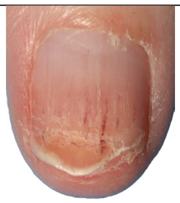	Linear dark lines	4 =YES		
9. Infection	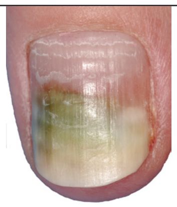	Swelling, pus, seeping fluid	6 =YES		
10. Nail bed separation<br>• **partial loss**	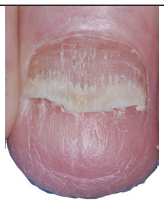<br>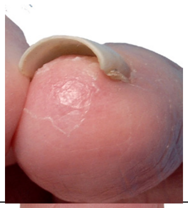	Part of nail missing<br>or<br>Nail plate partly lifting	8 =YES		
11. Nail bed separation<br>• **total loss**	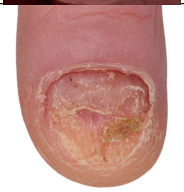	The entire lifting/ shedding of the nail plate from the nail bed	10 =YES		
12. Pain in nail bed	The individual's description of pain felt on or around the **nail bed / plate** (not peripheral neuropathy)		0 = None<br>2 = Mild<br>4 = Moderate<br>6 = Severe		
	Add all sections 1-12 in last column.			TOTAL OVERALL SCORE =<br>Maximum Possible Score = 524	
